# Recent advances in Shrimp aquaculture wastewater management

**DOI:** 10.1016/j.heliyon.2021.e08283

**Published:** 2021-10-30

**Authors:** Benedict Terkula Iber, Nor Azman Kasan

**Affiliations:** a Higher Institution Centre of Excellence (HICoE), Institute of Tropical Aquaculture and Fisheries (AKUATROP), Universiti Malaysia Terengganu, 21030 Kuala Nerus, Terengganu, Malaysia; b Department of Fisheries and Aquaculture, Federal University of Agriculture Makurdi, P.M.B. 2373, Benue State, Nigeria

**Keywords:** Shrimp, Aquaculture, Wastewater, Environment, Public health

## Abstract

Aquaculture has been celebrated globally and believed to usher in a viable alternative to capture fisheries. It is most welcomed especially now that the world population explosion has pushed the demand on fisheries products to worrisome limits. Shrimp farming is an area of aquaculture that has witnessed significant growth in recent years, contributing substantially to the global aquaculture production. However, intensification of shrimp aquaculture has come with unintended consequences such as wastewater management and other problems emanating from environmental impact of the wastewater. This study identified excess feed and fertilizer application, metabolite wastes, shrimp mortalities, oil spillage from farm machines, drug and chemical abuse as some of the activities contributing to wastewater generation in shrimp aquaculture farming. The impact of shrimp effluent water discharged has been observed to be socio-economic with both positive and negative dimensions. In attempt to overcome the overwhelming problems associated with shrimp effluent water and bring reassurances to its sustainability, a good number of new technological approaches have been identified including caviation, high-rate algal pond system, use of nanomaterials, biofloc technology, nanoadsorbent and polymeric nanoadsorbents. Although all have been proven to be useful, none could boast of a complete and integrated approach that considers all the technological, legal, social, environmental, public health and institutional concerns.

## Graphical abstract

**Figure:**
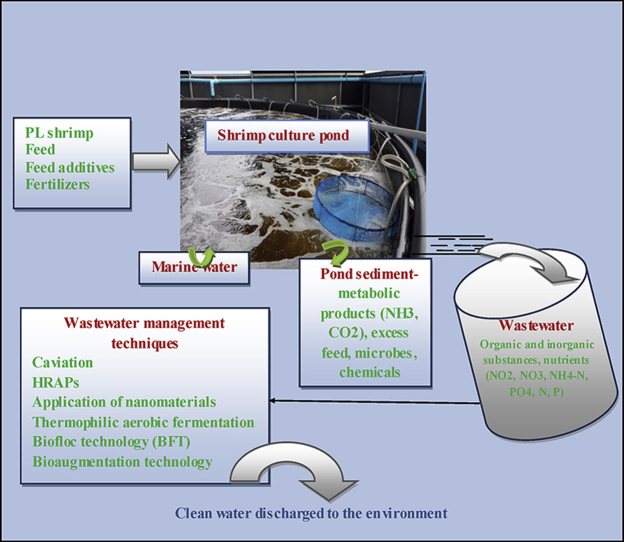

## 1 Introduction

Unless modern and better ways of handling aquaculture operations are adopted, “tragedy of the commons” is inevitable as far as environmental impact is concerned. Undoubtedly, aquaculture poses as the fastest industry worldwide with enormous potential to arrest the ever-increasing wild fish demand thereby bringing a steady halt to overfishing. The consumption of fish is estimated to hit one million tons globally by 2030 (Rajeev et al., 2021). This has shifted attention to marine food especially in the world trade market. The major aquatic organisms gaining world recognition even at the international market are shrimps, salmon and bivalves. This group altogether make up over 150 billion USD in the yearly international fish and fishery product trade (Patil et al., 2021). The world yearly shrimp production went up to about three million tons in the last 20 years. This was made possible from the intensification of shrimp culture by way of high stocking density and application of quality proteinous diets. It has been reported that feed meant for shrimp contain on the average of 30–40 percent crude protein of which only about 20–25 percent is utilized by the shrimp leaving the rest at the pond bottom as organic waste. Research has shown that up to 50 g of ammonia nitrogen is produced for every one kilogram of shrimp feed intake. In addition, a few other activities such as shrimp feces and dead organisms also add ammonia (NH_3_), nitrite (NO_2_) and hydrogen sulphide (H_2_S) to the pond water, making water unsuitable for recycling (Iber et al., 2021).

Water pollution, biodiversity loss, disease outbreaks and habitat destruction resulting from the build-up and discharge of metabolites from the shrimp culture facilities are a major factors hampering high productivity in the system. These do not only affect productivity but also impact negatively on the environment thereby promoting clashes between shrimp aquaculture and other ventures. This has greatly undermined the sustainability of shrimp aquaculture and hence, a call for measures to mitigate this deterioration trend. Various attempts have been expressed to reduce the nutrient content of shrimp aquaculture wastewater at the laboratory and field scale. The wastewater treatment can be on-site bioremediation or out-site treatment (Ni et al., 2021). On-site bioremediation refers to the natural wastewater treatment carried out at the point where it is generated. For instance, treatment of shrimp wastewater inside the pond during culture period; while out-site treatment entails the treatment of effluent water that has been moved out of the contaminated site.

The urgent need to come up with useful advances to tame the serious negative impacts of shrimp aquaculture wastewater is compelling and can never be overemphasized. It has been reported that over 1.2 billion people are affected by poor water quality resulting to about 15 million death in children yearly over the world (Ni et al., 2021). As a result of these staggering figures of death resulting from water pollution, many conventional methods have been advanced for shrimp aquaculture wastewater management. Some of these include coagulation, advanced oxidation process, membrane filtration process, adsorption, dialysis, phytocatalytic degradation and biological methods (Li et al., 2019, Möller et al., 2020). These methods have been proven effective in the removal of toxic substances causing pollution in shrimp aquaculture and other wastewater generating ventures. This study focuses on shrimp aquaculture wastewater generation and impacts on the environment. It also attempts to highlight some of the technological advances made in recent years to address the challenges posed by wastewater management in shrimp farming.

## 2 Activities leading to wastewater generation in shrimp aquaculture systems

In shrimp farming, wastewater may be generated during the full harvest stage. During production, shrimp feed, feces and dead organisms may significantly pollute the water (Ge et al., 2019). In another scenario, excessive use of chemicals in the culture system as well as disturbance of the pond bottom sediments can also pollute the upper layers of the pond water (Tampangallo et al., 2020). In addition, poor farm management practice such as littering of the farm with wastes resulting from dead shrimps can also add to the pollution load into the cultured water.

### 2.1 Feed and fertilizer application

Shrimp aquaculture relies mostly on external nutrients supply either on-farm formulated feeds or bought from commercial suppliers. The significant amounts of uneaten and undigested feed resulting from shrimp feeding accumulate at the bottom of the pond and become decomposed by microorganisms (Thornber et al., 2010). This activity increases the biological oxygen demand (BOD) of the pond water. Fertilizers are introduced in shrimp ponds to promote primary productivity (Hlordzi et al., 2020). However, fertilizers and shrimp feeds contain larger levels of nitrogen and phosphorous than the actual culture water. Nitrogen and phosphorous are the principal elements that promote eutrophication in aquaculture wastewater resulting in ecosystem destruction.

Report has shown that over 8.2 tons of chemicals and 41.7 ton products of biological origin are being applied together with feed supplements in intensive and semi intensive shrimp culture. Other frequently used compounds in the system were identified to be feed additives, soil and water treatment compounds, antibiotics, pesticides and disinfectants (Lyle-Fritch et al., 2006). While feed additives, add to the nutrient load of the wastewater, antibiotics, disinfectants and pesticides carry dangerous elements that are retained in water as residue and persist for a long time in the environment when discharged.

The application of nitrogen (e.g. urea) and phosphorus (supper phosphate) based fertilizers in shrimp aquaculture system is mainly to encourage primary productivity. However, this is accompanied by associated environmental impacts. These impacts are determined through some effect indicators. Some of the indicators include biological oxygen demand (BOD), chemical oxygen demand (COD), total suspended solid (TSS), total nitrogen, total phosphorus, N–NH3 and total coliform content of the wastewater. More often than not, nitrogen and phosphorous have been identified as the culprit elements contained in fertilizers which encourage eutrophication in the environment when in excess amounts. Report has shown levels of ammonia nitrogen, total nitrogen and total phosphorus levels of up to 1.8, 1.6 and 1.7 mg/L respectively in shrimp aquaculture wastewater (Anh et al., 2010).

### 2.2 Metabolic wastes

Feces are a major waste resulting from feed digestion in shrimps. The release of feces into the culture water, no doubt contributes to the pollution load of the water. Shrimp feces are rich in nitrogen and phosphorous which causes excessive algae growth, which potentially to cause algae bloom in water bodies receiving effluents from shrimp farms (Jasmin et al., 2020).

The heavy feed taken by shrimp in intensive culture system undergoes metabolism thereby releasing toxic nitrogenous substances into the culture water. These substances initiate series of reactions leading to products that add to the pollution level and deteriorate water quality. The major metabolic wastes reported in shrimp aquaculture wastewater are ammonia, urea and carbon dioxide (Patil et al., 2021). As reported in the culture of *Peneaus monodon,* addition of these wastes into the culture water comes in two ways; either from the digestion and metabolism of egested feed or from unconsumed feed which make up about 11% of the total feed applied. Among the metabolic products reported from shrimp wastewater, ammonia has been identified as the most toxic and a major challenge in wastewater management. High total ammonia nitrogen has been reported to hamper shrimp production by lowering the water quality. To overcome this, regular water exchange is advised. However, apart from the laborious nature of water exchange, where to discharge the old water is often the problem. Therefore, a better way of handling such high ammonia level wastewater is to lower the ammonia level by chemical or biological treatment before discharge into the environment (Lyles et al., 2008).

The connection between ammonia, nitrite and nitrate has also been reported under nitrogen cycle. Ammonia is first converted to nitrite by nitrosomonas and nitrococcus bacteria and then finally to nitrate. Naturally, ammonia and nitrate occur in moderate amounts in water, nevertheless, organic matter production by autotrophs relies basically on the presence of ammonia and nitrate. In shrimp aquaculture, acceptable concentration of unionized ammonia nitrogen is 0.0125 mg/L and considered toxic at levels higher than 1.5 mg/L (Roy et al., 2010). High excretion rate, increased level of ammonia in the blood and tissue, high blood pH and decrease oxygen consumption by tissue leading to gill damage are some of the toxic effects of ammonia. The toxicity of nitrite as a metabolic product in aquaculture wastewater has been reported at 0.2, 2 and 4 mg/L at acidic pH. Reaction of nitrite to blood hemoglobin to form methemoglobin is known to affect the oxygen carrying capacity of the culture organism. On the other hand, toxic effects of nitrate to aquatic organism have been reported as increase susceptibility of the affected organism to disease infection, low fertility and survival. Lethal concentration of nitrate has been reported at 3400 mg/L in *Penaeid shrimp* (Md. Yusoff et al., 2011).

The nutrient load of shrimp aquaculture wastewater has been estimated as the difference between the nutrients contained in the feed and that which is retained by the biomass. The loss of nitrogen and phosphorus into the culture waster has been estimated at 89% and 102% respectively (Verdegem, 2013). However, the amount of nitrogen and phosphorus losses depends on the type of species cultured or the production system. For instance, 46 kg load of nitrogen has been reported per metric ton of culture organism; while 14.4 kg was realized for phosphorus. According to Cao et al. (2007), an experiment in a shrimp farm at Guangdong province, China showed that shrimp wastewater contained 2.8% N and 1.8% COD which are often discharged into the environment. Although the global contribution of N and P by shrimp aquaculture wastewater is relatively small compared to other sources, the impacts of eutrophication emanating from the contribution cannot be overlooked.

### 2.3 Shrimp mortalities

Mortalities in shrimp culture are inevitable especially when there is poor water management. This results to poor water quality and disease outbreaks. Dead shrimps, especially when allowed to remain in the water column for a long time decays and add to the organic load at the bottom of the pond. The decomposition of this organic matter creates anoxic conditions in the polluted water (Prachumwat et al., 2020).

The amount of organic matter generated from shrimp mortalities and other waste products in intensive cultures have been reported to be largely influenced by the level of crude protein content of the feed and feed conversion ratio (FCR). For instance, at 1.2 and 1.5 FCR of 40% crude protein diet, 48 kg and 70 kg of organic wastes were generated per ton of shrimp production (Badraeni et al., 2020). The high levels of inorganic nitrogen compounds produced from the decomposition of this organic waste are known to be toxic to shrimp and results to mass mortality. Dead shrimp in culture water which further pollute the water is finally discharged into the environment. Statistics from world crustacean aquaculture has shown that the sub-sector discharges up to 3.74 × 10^10^ m^3^ of effluent to the environment. This report further showed specifically that shrimp aquaculture alone contributes 5,345–7157 cm^3^ of effluent for every ton of shrimp produced (Ng et al., 2018). Although these data may give an estimate of the level of shrimp effluent discharge, the actual situation may be far off since local shrimp producers are not always captured.

The impact of the organic matter in shrimp effluent on the coastal environment and its influence on nitrogen (N) and phosphorus (P) load has also been studied (Martínez-Durazo et al., 2019). The N and P discharged may come from intensive or semi-intensive culture systems and often in form of ammonium, nitrite, nitrate and phosphate. The nutrient levels of shrimp culture water have been reported as shown in Table 1. The constant discharge of organic matter to the environment should be a source of worry and a focus for conservation efforts. Better farm management efforts such as closed system and better feed efficiency and support to local shrimp farmers have been suggested to address the problem of organic matter discharge into the coastal environment (Hargan et al., 2020). Closed system for instance helps to retain organic matter and nutrients before wastewater discharge from the shrimp culture facility. By so doing mangroves can flourish next to shrimp aquaculture where nutrients in the pond effluent are attenuated before getting to the coastal environment.

**Table 1:** Nutrient levels of shrimp aquaculture wastewater.

Nutrient	Symbol	Level	Culture system	Days	Reference
Ammonium	NH4^+^	0.60 0 ± 0.370 mgL^−1^	Semi-intensive	10	Alfiansah et al. (2018)
Ammonia	NH_3_	0.181 ± 0.012 ppm	Intensive	30	Badraeni et al. (2020)
Nitrite	NO_2_^-^	0.201 ± 0.334 mgL^−1^	Intensive	40	Alfiansah et al. (2018)
		0.446 ± 0.376 ppm	Intensive	30	Badraeni et al. (2020)
Nitrate	NO_3_^-^	0.213 ± 0.184 mgL^−1^	Intensive	40	Alfiansah et al. (2018)
		1.963 ± 1.693 ppm	Intensive	30	Badraeni et al. (2020)
Phosphate	PO_4_^3-^	0.72 ± 0.07 mgL^−1^	Intensive	40	Alfiansah et al. (2018)

### 2.4 Oil spillage

The commercialization of shrimp aquaculture requires the use of machineries like generators, automated feeders, water pumps, aerators, outboard motors, vehicles and lawnmowers. The fuels and lubricants used in running or maintaining these machines may spill and wash into shrimp ponds causing pollution. Spillage may occur because of negligence, operations, servicing or repairs.

### 2.5 Drugs and chemicals

Shrimps, unlike other fish species, do not have acquired immune system and can therefore be more prone to pathogens and do not respond to vaccination. Drugs and chemicals are utilized by shrimp farmers in preparing the culture facilities, growth promotion and treatment of diseases. Most common drugs and chemicals used include salt, lime, potassium *per-manganate, malathion, formalin, sumithion, malachite green* and bleaching powder. A few other antibiotics include *co-trimoxazoie, oxysentin, oxytetracyline, renamox, sulphadiazine, chlorotetracycline, renamycine, amoxicillin and orgamycine* (Thornber et al., 2020). These chemical substances have been implicated in several ways as contributing to pollution in shrimp aquaculture.

Antimicrobial resistance (AMR) is an expanding threat to the environment. AMR refers to a situation where disease causing microorganisms develop resistance for drugs specifically designed to kill them (Lee et al., 2019). This is highly common among the low- and middle-income shrimp farmers where there is abuse of the use of antibiotics. It is difficult to assess the level of abuse of these drugs at present because of lack of surveillance and paucity of data.

## 3 Impacts of shrimp aquaculture wastewater on the environment

Shrimp aquaculture wastewater impacts the environment in many ways. In most cases, the negative impacts are the most projected. The management of shrimp effluent water yielded significantly to the production cost in terms of operational and extra capital cost (Chatla et al., 2020). Also, the cost for environmental protection to ensure good public health combines with the already compounded problems shrimp aquaculture wastewater handling. Shrimp aquaculture effluents also have some positive impacts to the environment; some of which are as discussed below:

### 3.1 Positive impacts of shrimp aquaculture wastewater

#### 3.1.1 Economic benefits

Shrimp aquaculture wastewater if properly managed could be utilized by farmers at no cost to enhance pond nutrients to increase productivity. Shrimp farmer can obtain wastewater free of charge and may only incur little cost of treatment to make useful for shrimp rearing. No supplementary fertilizer is needed to improve primary productivity, and in some cases, supplementary feed may not be required thereby lowering production cost (Viegas et al., 2021). Shrimp farmers may also combine crop production with shrimp farming, with the establishment of the adjacent parts of the pond where wastewater rich in nutrient is used for irrigation. This helps to create employment and additional income for the farmers.

#### 3.1.2Environmental benefits

Shrimp farms that are well managed would certainly have some important effects on the environment receiving its wastewater. Some of these include:

i. Shrimp wastewater recycling helps to mitigate environmental degradation and water conservation. The conservation aspect leads to a more rational use of natural water thereby preventing wastage.
ii. The problems of wastewater encourage farmers into research for better wastewater handling ways. This leads to the development of low-cost wastewater treatment techniques and new advances to convert effluent water into resource.
iii. The release of wastewater into the environment may lead to complete change of the biota from less useful to more beneficial one.
iv. Shrimp aquaculture wastewater is also useful in recovery of poor sandy soils which are known to be highly deficient in plant nutrients.
v. Wastewater from shrimp farms if properly managed is useful for irrigation purposes for higher crop productivity.
vi. The increase in plant diversity of the wetlands receiving nutrient rich shrimp aquaculture wastewater may give birth to good variety of trees which are useful for timber purposes.

### 3.2 Negative impacts of shrimp aquaculture wastewater

#### 3.2.1 Effects on public health

Shrimp farmers and consumers exposed to toxic wastewater stand the risk of health challenges. Shrimp effluent water habours bacteria, viruses and many other forms of disease transmitting parasites (León-Cañedo et al., 2019). These microorganisms aid in disease transmission among shrimp handlers and the communities at the receiving end of wastewater discharge. The constant exposure of shrimp farming communities to diseases leads to a reduction of the labour force and their earnings, generally resulting to impoverishment from exorbitant medical bills.

In recent years, the impacts of shrimp aquaculture wastewater have been proven to extend beyond the influence of excess nutrients on eutrophication and alga bloom (Md. Yusoff et al., 2011). Shrimp wastewater also contributes to ozone layer depletion by emission of nitrous oxide (N_2_O) and methane (CH_4_) to the atmosphere where discharged untreated. A study on shrimp effluent water in Nansha County, China showed that mangroves receiving shrimp wastewater were able to eliminate nitrite (NO_2_), nitrate (NO_3_) and ammonium ions (NH_4_^+^) with efficiency of 43.6%, 41.2% and 65.0% respectively. However, results indicated that mangroves receiving shrimp wastewater had 2 to 3 times higher levels of CH_4_ (0.695 mgL^−1^) and 3 to 9 higher levels of N_2_O (0.493 mg L−1) than wetland without shrimp effluent water (Wang et al., 2021). The results of this study prove the fact that whereas mangrove wetlands are capable of absorbing the excess N and P from the shrimp effluent water, their inability to prevent greenhouse gases emission has made it imperative for proper wastewater treatment before discharge.

#### 3.2.2 Soil degradation

The wastewater collected from shrimp aquaculture farms are rich in nitrogen, phosphorous, salts, heavy metals and many other toxic substances. These substances are in most cases found in excess amounts in the effluent water. Therefore, their continuous use for irrigation and other agricultural purposes may lead to long term negative impacts on the agriculture soils. Crops reared in soils with excess nitrogen and phosphorous grow vegetatively to the detriment of production; while heavy metals in the soil are taken up by plants and subsequently consumed alongside with plants by other organisms including human (Krishnani et al., 2018).

Apart from excess nutrients, shrimp effluents have other impacts on the local environment including agriculture soil. For instance, elevated soil salinity has been reported around coastal environments receiving wastewater from shrimp farms. The level of salinity becomes higher as one gets closer to soils near shrimp ponds. In specific terms, every one meter decrease in distance between the shrimp culture facility and the adjacent land induces a 0.14% higher salinity of the soil according to Johnson et al. (2016). It has also been estimated that for every 10% increase in soil salinity from shrimp farms results to 0.6% decrease in paddy yield in south India (Morshed et al., 2020). This has indeed increased the externality cost of shrimp aquaculture and may lead to social conflicts.

In another study, three mangrove sites known to have received nutrients from shrimp aquaculture for over a period of 0–14 years were tested for effect of salinity on total organic carbon (TOC), total nitrogen (TN) and total phosphorus (TP). Report showed that shrimp wastewater increased soil TOC, TN and TP significantly. The shrimp effluent contributed 30.00%–33.60% of the coastal soil TOC up to 10 cm deep (Tian et al., 2019). This effluent discharge also change the carbon and nutrient pattern of the receiving area. Over 50%–90% of carbon has been reported to be stored in the soil (Ahmed et al., 2017). It has become necessary to understand the level of carbon storage in coastal environment for better global carbon sequestration.

#### 3.2.3 Impact of shrimp aquaculture wastewater on biodiversity

**Figure 1:**
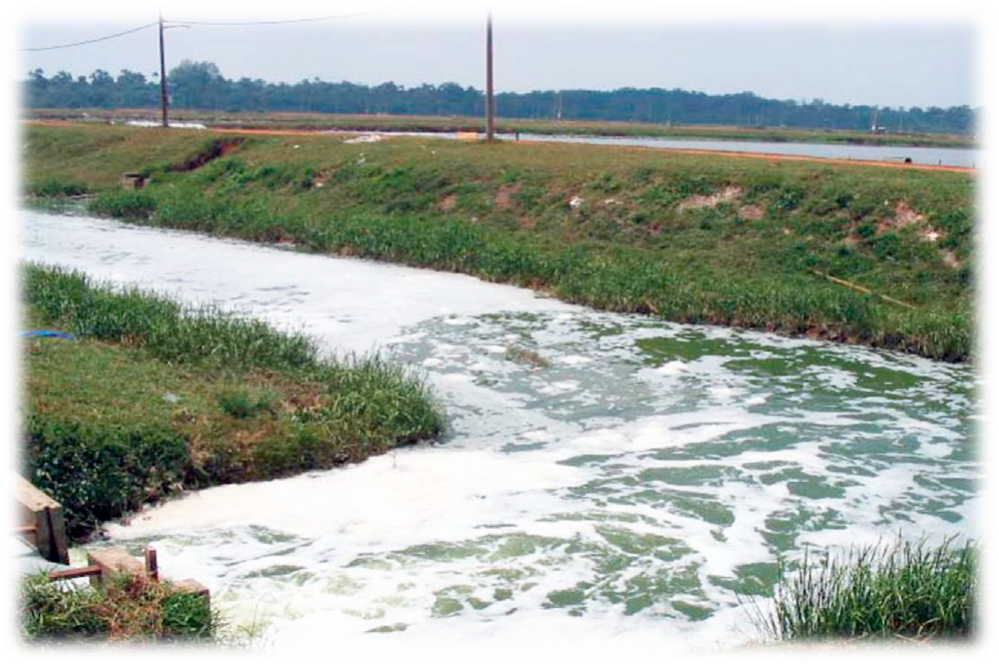
Wastewater discharge from a shrimp farm, modified from Hargan et al. (2020**)**.

The incessant discharge of high nutrient shrimp aquaculture wastewater to the adjoining environment (Figure 1) affects the biodiversity of creeping, swimming and flying organisms of such areas. The nutrient rich and toxic effluent water destroys the breeding sites, nesting beds, roosting grounds and bird shelters (Khan et al., 2021). In addition, more useful biota may be destroyed giving way to less important but more tolerant species of organisms. The toxic shrimp wastewater also impacts so much on the biota leading to the total extinction of some organisms.

#### 3.2.4 Social impacts of shrimp aquaculture effluent water

Some of the social challenges posed by discharge of shrimp aquaculture wastewater stem from the nuisance they create. The offensive smell, environmental degradation and lack of proper hygiene are some the social issues arising from the shrimp aquaculture operations. Continuous discharge of this waste leads to food safety problem, poor quality health, loss of property value and reduction of life expectancy of the community in the long run (Hang Pham et al., 2021). As a result of these impacts, people are forbidden from consuming aquatic organisms from natural environments perceived to be polluted with aquaculture wastewater. Furthermore, shrimp farmers involved in wastewater aquaculture have difficulty in selling their harvested shrimp since many would only choose for shrimps reared in clean water (Xuan et al., 2021). This does not only affect the prices of the shrimp but also impact negatively on the income and welfare of the farmers. However, farmers in this category may tend to process the shrimp before transporting to the market since cooked shrimp are perceived to be less contaminated.

Studies have shown that shrimp aquaculture effluent can contribute to making or loosing commons depending on the multilevel driver's activities. Some of the activities of multilevel drivers contributing to lost of common include large scale production, individually owned shrimp farming operations, encroachment of customary fishery commons, loss of commons access and entitlements, breakdown of commons institutions, policy changes, caste politics and resource conflicts, ecological disturbance and general changes in fishery practice. On the other hand, a good number of factors influencing the making of commons in shrimp aquaculture operation have also been outlined. These include but not limited to: coordinating discharge, built-in incentive for stewardship, multilevel commons institutions, collective decision making, bottom up management approach, mixed common regime and small scale shrimp farming operations (Béné et al., 2016). Expansion of coastal shrimp aquaculture leads to the displacement of small scale fishers who earlier inhabited such areas and depending on it for their livelihood. This also leads to socio-economic problems such as mass migration to urban areas, marginalization, and rural unemployment, and food insecurity, loss of institution, social unrest and conflicts. A study at Chiliki Lagoon in the Bay of Bengah India showed that out of 140 fishing villages surveyed, up to 135 fishers complained of being adversely affected by shrimp aquaculture operations in the area (Galappaththi and Nayak, 2017).

## 4 Recent technological advancements in shrimp aquaculture wastewater treatment

Nature has created a self-regenerating system for water use and availability through a process called the water cycle. Nevertheless, it becomes extremely difficult to have sustainable and good quality of water in constant supply due to over-use. Water over-use especially for aquaculture purposes has impacted the sustainability of water negatively over time. Shrimp aquaculture wastewater treatment has attracted so much attention in recent years due to increase water demand, giving rise to several advances in water treatment techniques.

### 4.1 Caviation

Caviation is a method of wastewater management that utilizes the effect of temperature and pressure on cavities filled with gas called bubbles (Mancuso et al., 2020). Using a mechanical device to initiate pressure pulses, the bubbles inside the cavity continue to expand until the vapour pressure of the liquid equals the external pressure. The threshold reached at this point forces the bubbles to burst. Reports have shown that bubble burst could increase the inner temperature and pressure above hundreds. This higher temperature pressure leads to the splitting of water into hydrogen and hydroxyl ions. Due to the strong oxidative nature of the hydroxyl radical, it quickly oxidizes the chemical toxicants in water. The oxidation reaction also helps to remove pathogenic microorganisms from the shrimp culture wastewater (Xuan et al., 2021).

Caviation has proven a few advantages over the usual advanced oxidation process for wastewater treatment. One of such advantage is that it does not require reactants and ultraviolet light for its operation (Joshi et al., 2019). This alone helps to reduce the cost of water treatment. Another advantage of caviation is seen in the fact that by-products are minimal as compared to other techniques. Thus, the by-products are limited to the contaminants in the wastewater.

In caviation, aside being the best method for oxidizing organic contaminants, the collapse energy of the system facilitates the complete destruction of the cell structure of bacterial and other microorganisms in the wastewater. This has been shown to be more effective in drying of biomas and biogas production thereby enhancing wastewater treatment operations (Tandiono et al., 2020). Results have shown that hydrodynamic caviation can disrupt over 90% bacteria *Escherichia coli* in 5 min. Relatedly, over 94% of pathogenic bacteria *Microcystis aeruginosa* have also been disrupted (Gągol et al., 2018). With this advancement, the nutrient rich shrimp effluent water could be made safe from disease causing organisms before discharge into the environment. The combination of caviation and Biofloc technology in the festival-style shrimp farming method was also able to remove chemicals, shrimp feces and fed debris successfully from the wastewater (Kwon et al., 2021).

### 4.2 Use of high-rate algal pond system (HRAPs)

High-rate algal ponds (HRAPs) have been modified for treatment of shrimp aquaculture wastewater and effluents from other sources. These are shallow ponds with less than one meter depth, attached with paddle wheel for horizontal water movement. Water could be set into motion up to a speed of over 0.2 m/s. Carbon dioxide (CO_2_) is injected inside the counter current gas sparging sump usually laid below the paddle wheel (Robles et al., 2020). This arrangement creates a turbulent water movement within the pond. High level algal growth is allowed in this pond after which they are harvested for processing into other useful products such as biofuels. It is believed that the algae would take up the excess nutrients from the polluted water which then be recycled or discarded into the environment (Hargan et al., 2020). One advantage of this technique is that algae could be converted into other commercially important materials that would add income to the farmer. However, the shortcoming lies in the difficulty selecting the suitable type of algae for culture.

The use of alga pond system in shrimp aquaculture wastewater management is effective, highly profitable and indeed a green technology. In a study conducted by Soka University Japan, using a simulated aquaculture wastewater, HRAPS were able to remove 100% of the nutrients in form of ammonium, nitrate and phosphate; whereas, over 80% organic matter were also successfully taken out (Kishi et al., 2018). This result did not only prove the effectiveness of HRAPs for high aquaculture wastewater treatment performance but also the valorization of algal biomass produced. In another report, *Arthrospira sp.* and *Nostoc sp.* PCC7413 were selected as the algal species for nutrient removal from wastewater. Results showed ammonium efficient removal of 84.9 ± 1.9% by *Arthrospira sp.* and as low as 4.9 mg/L ammonium concentration in the treatment with *Nostoc sp.* PCC7413. This level of removal efficiency in both algal species could be achieved in 24 hours (Álvarez and Otero, 2020).

The application of plants in shrimp effluent water treatment through a process called bioremediation has been in the fore in recent times. More plants have proven successful in absorbing excess nutrient from shrimp wastewater before discharge. In an integrated culture involving shrimp cum mussel cum aquatic macrophyte algae in Romania, higher efficiencies in the removal of pollutants were clearly demonstrated by the algae. In specific terms, 29%, 79%, 76% and 99% of COD, suspended matter, total nitrogen and total phosphorus were removed respectively (Tociu et al., 2017). The benefits of using aquatic plants in the treatment of shrimp wastewater go beyond the success in pollutant removal. For instance, Elizondo-González et al. (2018) in their feeding trial involving *Ulva lactuca* and shrimp culture, the plants were harvested, dried and ground to formulate fish feed. Furthermore, properly treated shrimp effluent water will not only prevent environmental degradation but also reused in aquaculture or other purposes, thereby ensuring proper management of water resource. This has become important now than ever in the wake of human population explosion. The application of shrimp wastewater treatment system consisting of agar-alginate blocks was investigated where results showed that higher removal rates of phosphate, nitrate, nitrite and ammonia by *Picochlorum maculatum* in the system were 57%, 46.4%, 89.6% and 98.5% respectively (Kumar et al., 2016).

### 4.3 Use of nanomaterials for shrimp aquaculture wastewater treatment

Nanomaterials are those materials with particle size ranging from 1–100 nm. These materials can be grouped according to their size, morphology, physical and chemical characteristics (Do et al., 2019). A good number of nanomaterials are carbon-based, ceramic, metallic, semi-conductors, polymeric and lipid based in nature (Ighalo et al., 2021). Nanomaterials are used in various forms for different ways achieving wastewater treatment to enhance environmental protection. Some of these forms are as follow:

#### 4.3.1 Nanoadsorbents

These materials are used to accomplish shrimp aquaculture wastewater treatment through adsorption process. The process involves the extraction of all forms of pollutants from the effluent water by attracting to the active sites on the outer surface of the adsorbent material all the pollutants in the water (Manyangadze et al., 2020). This method has been proven to be better than the normal adsorption method, having better surface chemistry and shortest time intra-particle diffusion distance. In addition, nanoadsorption technique has also exhibited higher potential to the conventional adsorbent method where it has demonstrated higher specific surface area for contaminant binding and more associated sorption sites.

#### 4.3.2 Use of polymeric nanoadsorbents

In recent years, some nanomaterials have been fashioned for specific pollutants. These pollutants may be organic, inorganic or basically for some targeted heavy metals. Polymeric nanoadsorbents may be made in such a way that the inner part of it is water repelling to adsorb only organic materials whereas the outer part is specifically made to attract hydroxyl or amine groups of pollutants (Thamer et al., 2021). These forms of nanomaterials are also called dendrimers. This particular design of polymeric nanoadsorbent is effective in taking up heavy metals via complexation, electrostatic interaction, hydrophobic effect and hydrogen bond.

#### 4.3.3 Use of nanomaterial-based membrane

The lifetime and effectiveness of a membrane in shrimp aquaculture wastewater treatment system is dependent on its energy need, the ease in maintaining the selectivity of the membrane, permeability and whether it is prone to biofouling (Zhang et al., 2019). Recent approaches to increase the permeability of membranes used in wastewater treatment using nanomaterials have shown great results. This has proven to not only improve permeability but also increase the fouling resistance, mechanical and thermal stability.

Membrane technology is introduced in the conditioning of shrimp culture water and effluent water treatment before eventual discharge into the environment. it is also applied in the recovery of nutrients from the shrimp aquaculture wastewater for further agronomic uses. The use of membranes of the range between 0.1–10 μm pore sizes in shrimp farming has been proven to be better in terms of ease of cleansing; which can easily be achieved through back-flushing. The ability of membranes to remove viruses, sludge and phosphorus has also be reported (Ng et al., 2018). This has further placed them as better options for the treatment of high nutrient rich shrimp aquaculture wastewater. The use of nanomaterial-based membrane in shrimp aquaculture wastewater management is still an emerging technology. Although very little studies have been performed so far, huge potentials associated with the method have no doubt made it a viable alternative.

#### 4.3.4 Use of nanofiber membranes

A typical problem with the conventional membranes used in wastewater treatment is the difficulty to manipulation. The incorporation of nanofibers to membranes in recent times has given rise to membranes with higher specific surface areas and greater porosity. These nanofibers incorporated membranes can be altered in terms of their diameter, morphology, composition, secondary structure and alignment (Cui et al., 2020). Reports have shown that these membranes have low fouling tendencies with high capability of adsorbing small particles from an aqueous phase at a high rejection rate.

In a study to determine the effectiveness of nanofibers reactors on the removal of nitrate and phosphate in aquaculture wastewater, nanofibers particle successfully decreased nitrate and phosphate by 70.52 and 70.48% respectively. The dangers of excess nitrate and phosphate to the natural aquatic habitats receiving aquaculture wastewater are no longer in doubt. Studies have shown that about 0.4 mg/L of nitrogen and 0.1 mg/L of phosphorus are capable of supporting algal bloom. Nanofiber particles have so far shown excellent results in the treatment of phosphate, nitrate and dissolved oxygen. In a study that lasted three weeks, nanofiber particles successfully reduced nitrate concentration form 48.61 ± 7.2 mg/L to 16.01 ± 9.6 mg/L while phosphate decreased from 8.52 ± 1.27 mg/L to 2.86 ± 0.47 mg/L at the third week. In the same study, DO and pH were also corrected from 5.17 ± 2.18 mg L to 4.83 ± 1.62 mg L and 8.69 ± 0.1 to 8.73 ± 0.19 respectively (Askari Hesni et al., 2019).

### 4.4 Solid state thermophilic aerobic fermentation for nutrient recovery from shrimp wastewater sludge

This technique is born out of the problems associated with sludge handling during wastewater treatment. Previous attempts have been made in this regard using the nutrient rich sludge to culture microalgae. However, the alga products from this arrangement have been reported to have limited applications due to the inability to produce algal materials free from contaminants (Shen et al., 2019). This singular disadvantage has restricted the use of the algae materials produced from this method to bio-energy purposes.

Solid state thermophilic aerobic fermentation is a new technique design to produce clean nutrient in form of ammonium gas from shrimp wastewater sludge for culture and harvest of algae free from pathogens and heavy metals (Koyama et al., 2018). In this method, the organic nitrogen of the wastewater is firstly broken down by microorganisms to dissolved nitrogen. The dissolved nitrogen is later transformed into ammonium nitrogen (NH^4+^ –N). Part of this is given off as ammonium gas. The pure ammonium gas which is devoid of contaminants such as heavy metals and pathogens becomes the source of nitrogen for micro algae production.

In a study involving combination of fermentation and microalgae production in marine aquaculture wastewater, *Chlorella vulharis* (*C. vulgaris*) growth were highest at 25 °C and removal efficiency of COD, ammonium nitrogen were 94.4 and 68.8% respectively. The result from this combination proved the environmental sustainability and economic feasibility of the technology in shrimp aquaculture wastewater treatment (Zhang et al., 2021). Many other species of algae have shown similar or even higher performances in both aquaculture and other forms of wastewater. For instance removal efficiencies of 75.8% COD and 83.4% ammonia by microalgae have been reported in municipal wastewater. However, the difficulty in selecting the desired species for culture has limited the wide range application of this method (Chalima et al., 2019, Ren et al., 2019).

### 4.5 Biofloc technology (BFT) in shrimp aquaculture wastewater management

Owing to the ever increasing and serious environmental challenges bedeviling shrimp aquaculture farms especially in wastewater handling, a new technology has been developed. Biofloc technology (BFT) makes use of isolated biofloc boost-up bacterium inoculums to enhance better water quality of the shrimp culture in order to improve shrimp growth (Kasan et al., 2018; El-Sayed, 2021). This technology is a partial departure from the conventional biological filter used to remove ammonia, nitrate and dissolved organic solid in recirculation aquaculture system.

Although BFT presents a viable alternative for shrimp aquaculture wastewater treatment, some limitations such as the need for continuous aeration, constant waste removal and need for additional carbon source which is a major requirement for bacteria growth. However, latest advances have incorporated microorganisms, uneaten feed, detritus and suspended particles with water aeration to produce low cost biofloc which is rich in protein. The heterotrophic bacteria in the biofloc are capable of neutralizing ammonia in indoor tanks, an activity hitherto known to be carried out by outdoor microalgae (Olmos Soto, 2021).

Today, the push for green technology in wastewater treatment has become so strong as a result of the residual effects of chemical substances hitherto applied in the treatment processes. Consequently, bioremediation has gradually taken the centre stage. Numerous successes have been recorded in the use of biological processes for removal of excess nutrients such as N, P and ammonia from aquaculture wastewater (Lavania-Baloo et al., 2014). This has also been demonstrated on shrimp processing wastewater with resounding successes. However, the utilization of seawater has made biological process quite ineffective in the treatment of wastewater from shrimp processing factories. JEONG (2016) reported that removal of nutrients from shrimp processing wastewater can be better achieved through struviate crystallization by varying Mg^2+^:NH^4^–N:PO_4_–P in the molar ratio of 1:1:1 before the use of biological processes to remove organic matter.

### 4.6 Bioaugmentation technology

Bioaugmentation has been carried out using nitrifying and denitrifying microbial consortium to tackle the problem of nitrogenous metabolites in shrimp culture. This new technology intended to offer a green approach to shrimp aquaculture wastewater treatment incorporates ammonia oxidizing, nitrite and denitrifying bacteria to overcome the menace of total ammonia nitrogen in shrimp culture water (Patil et al., 2021, Shi et al., 2020). The microbial consortium has been reported to be stable at room temperature for up to 120 days, retaining its effectiveness.

In an attempt to reduce the high volume of water need for effective shrimp culture, the United State Marine Shrimp Farming Program (USMSFP) developed a technology known as recirculating raceway system which has proven to be effective in high quality shrimp yield at zero water exchange. Although this system helps to increase farmer yield and also conserve water, the high levels of ammonia, nitrite, and nitrate in the resultant wastewater has rendered the system environmentally unfriendly. Nevertheless, the application of sequencing batch reactor (SBR) in handling such nitrogen rich wastewater has been recommended (Lyles et al., 2008; Roy et al., 2010). The success of SBR is evident from the complete nitrification of ammonia and denitrification of nitrate, both aerobically and anaerobically in sequence. Studies have shown that at 10:1 of C:N in SBR and addition of molasses as carbon source, 99% of NH_3_, NO_2_ and NO_3_ have been removed successfully (Lyles et al., 2008; Boopathy, 2009; Roy et al., 2010). In another development, in situ hypochlorous acid (HOCl) oxidation of shrimp wastewater has been advanced. This process makes use of salinity present in the shrimp effluent water to produce HOCl.

Although studies showed a COD reduction of over 50 mg/L, the process of in situ hypochlorous acid (HOCl) oxidation is so complex and not cost effective (Vijayaraghavan et al., 2008). Many authors have argued that NH_3_ is by far the most dangerous component of aquaculture wastewater. This has prompted a lot of studies toward removing NH_3_ from the wastewater before discharge. It is for the same reason that Krishnani et al. (2006) utilized bagasse, a highly fibrous natural lignocellulosic by-product of sugarcane in the treatment of NH_3_ from shrimp aquaculture wastewater. Results showed that bagasse reduced NH_3_ concentration from 1.015 mg/L to 0.178 mg/L within 24 h. This was also followed by rapid decline in Total Ammonia Nitrogen (TAN) by up to 95%. Although this results are highly impressive, the efficiency of bagasse was found to be highly dependent on dosage applied, time and initial NH_3_ concentration.

## 5 Conceptual guideline for shrimp aquaculture practice

These refer to conscious efforts from shrimp farmers to prevent the negative impacts of wastewater and other forms of disasters on the farm (Pierrette Coulibaly et al., 2021).

i. Site selection: Shrimp aquaculture farms should be cited away from residential environments to avoid social impacts. Farmers should select sites that are wide enough to accommodate wastewater reservoirs for temporary storage and treatment.
ii. Stocking density: Good quality post larval (PL) should be used for initial stocking to avoid the incidence of disease and mortality. Attention must be paid at this point on the stocking density and carrying capacity of the culture facility. Overstocking will lead to poor water quality, poor growth rate and shrimp mortality.
iii. Food and feeding: Ensure good feeding practice to avoid excess feeding which leads to food wastage. Feed at a particular spot designated for feeding at pre-set times. Uneaten feed absorb water and eventually settle at the pond bottom. This later decompose adding excess nutrient to the water and cause oxygen depletion
iv. Shrimp health management: Always make provision for health facilities at the onset of the production. Farmers are advised to always look out for symptoms of disease and attend to them in time and appropriately.
v. Chemical and therapeutic agents: Chemicals such as antibiotics, fertilizers and drugs should be applied according to manufacturer specifications. Over application of antibiotics will lead to development of resistant strains of disease-causing bacteria in the culture system.
vi. Social responsibility: It is important for shrimp farmers to be in good communication and relationship with other land users close to them. Give assistance to local communities to show a good sense of responsibility, this will go a long way to minimizing the problem of social impact.
vii. Groups and training: Farmers should seek to obtain the basic knowledge on the management practices in shrimp farming. This can be achieved by formation of groups and associations to facilitate information and knowledge dissemination.
viii. Record keeping and data collection: Records on daily operations should be kept by the farmer for review purpose and auditing.

## 6 Conclusion

In the wake of global call for sustainable use of water, shrimp aquaculture farmers have no choice but to find ways of dealing with the menace of wastewater emanating from their production processes in order to remain in business. Effluent water from shrimp aquaculture must be treated to remove excess nutrients, especially nitrogen and phosphorous before reuse or discharge into the environment. The excess nutrients from the water may result from excess feed, fecal droppings of the shrimp and fertilizers. Furthermore, other toxic components of shrimp effluent water have been observed to emanate from oil spillage around the farm area, excess use of chemical substances and other poor management practices on the farm.

Due to the numerous socio-economic impacts of shrimp aquaculture wastewater, numerous attempts have been made to address the challenges. Some of the methods such as caviation, high-rate alga pond system, use of nanomaterials, solid state thermophilic fermentation, BFT among others have undergone several modifications in recent years to address specific challenges associated with wastewater treatment. It must be observed that for proper selection of an appropriate technology for shrimp aquaculture wastewater treatment, an integrated approach is required to bring into consideration all the technological, legal, social, environmental, public health and institutional aspects to take into consideration.

It is clear in this study that shrimp farming is indeed lucrative. However, due to the huge wastewater produce from the culture activities, shrimp culture should be carried out simultaneously with proper wastewater treatment technologies in order to protect the environment. In attempt to develop technologies for nutrient removal from shrimp aquaculture wastewater, excess use of chemicals must be discouraged. This is because chemical treatment, although removes nutrients from wastewater, their residual effects resulting from precipitation can linger in the discharged water for a long time causing further harm to the environment. Therefore, bioremediation is strongly recommended especially in this era of green technology. Bioremediation is not only eco-friendly but also provides better opportunities for income generation by the farmers. This is especially true where better plants species are selected and cultured alongside shrimps to absorb excess N, P and NH_3_ and are eventually harvested and processed into biofuel and animal feed.

## Declarations

### Author contribution statement

All authors listed have significantly contributed to the development and the writing of this article.

### Funding statement

This work supported by the Ministry of Higher Education (MOHE), Malaysia under Higher Institution Centre of Excellence (HICoE), Institute of Tropical Aquaculture and Fisheries (AKUATROP) program [Vot. No. 63933, JPT.S(BPKI) 2000/016/018/015 Jld.3 (23) and Vot. No. 56050, UMT/PPPI/2-2/5 Jld.2 (24).

### Data availability statement

No data was used for the research described in the article.

### Declaration of interests statement

The authors declare no conflict of interest.

### Additional information

No additional information is available for this paper.
